# Tolosa-Hunt Syndrome in Double-Hit Lymphoma

**DOI:** 10.1155/2015/249891

**Published:** 2015-03-30

**Authors:** Prakash Peddi, Kevin M. Gallagher, Chandrikha Chandrasekharan, Qi Wang, Eduardo Gonzalez-Toledo, Binu S. Nair, Reinhold Munker, Glenn M. Mills, Nebu V. Koshy

**Affiliations:** ^1^Department of Medicine, Section of Hematology and Oncology, Louisiana State University Health, Shreveport, LA 71103, USA; ^2^Feist-Weiller Cancer Center, Shreveport, LA 71103, USA; ^3^Department of Pathology, Louisiana State University Health, Shreveport, LA 71103, USA; ^4^Department of Radiology, Louisiana State University Health, Shreveport, LA 71103, USA; ^5^Department of Medicine, Division of Hematology and Oncology, Weill Cornell Medical College, New York, NY 10065, USA

## Abstract

Tolosa-Hunt syndrome (THS) is a painful condition characterized by hemicranial pain, retroorbital pain, loss of vision, oculomotor nerve paralysis, and sensory loss in distribution of ophthalmic and maxillary division of trigeminal nerve. Lymphomas rarely involve cavernous sinus and simulate Tolosa-Hunt syndrome. Here we present a first case of double-hit B cell lymphoma (DHL) relapsing and masquerading as Tolosa-Hunt syndrome. The neurological findings were explained by a lymphomatous infiltration of the right Gasserian ganglion which preceded systemic relapse. As part of this report, the diagnostic criteria for Tolosa-Hunt syndrome and double-hit lymphoma are reviewed and updated treatment recommendations are presented.

## 1. Case Report

**Figure 1:**
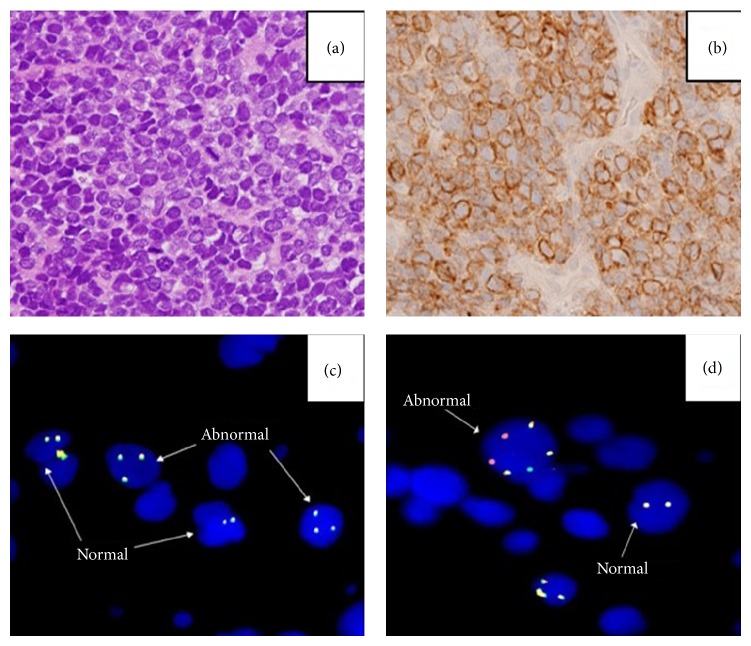
H&E staining demonstrating large B cells (a) positive for CD20 (b). FISH studies showing IGH-MYC translocation (c), dual color, dual fusion translocation probe identifying IGH and BCL2 gene rearrangements (d).

A 57-year-old Caucasian woman with no significant past medical history presented to our institution with painless hematuria, excessive fatigue, and twelve-pound weight loss for three weeks prior to presentation. Computerized tomography scan (CT) of the abdomen and pelvis revealed heterogeneously thickened urinary bladder wall with direct soft tissue infiltration into perivesicular spaces, left iliac adenopathy, omental implants, and an enlarged retroperitoneal node. Core biopsy of bladder revealed diffuse large B cell lymphoma of germinal center cell origin positive for CD20, CD79a, CD10, and MUM1 by immunohistochemical (IHC) studies. Molecular studies by fluorescence in situ hybridization (FISH) showed presence of IGH/MYC translocation and rearrangements of BCL2 giving the diagnosis of double-hit lymphoma (DHL) (Figure 1). Imaging with 18-fluorodeoxyglucose positron emission tomography and computerized tomography (FDG PET/CT) revealed heterogeneous FDG avid uptake involving urinary bladder, perivesicular soft tissues, and omental implants along with nodal involvement of inguinal, iliac, mesenteric, and retroperitoneal regions. Based on a diffuse infiltration of a parenchymatous organ (bladder) and weight loss a clinical stage IV B was determined.

Magnetic resonance imaging (MRI) of brain and cerebrospinal fluid (CSF) analysis were negative for lymphomatous involvement of central nervous system (CNS). Pending final pathology report, patient was initially started on rituximab, cyclophosphamide, doxorubicin, vincristine, and prednisone (R-CHOP) chemotherapy. After molecular studies confirmed diagnosis of DHL treatment was changed to dose adjusted rituximab, etoposide, prednisone, vincristine, cyclophosphamide, and doxorubicin (DA-R-EPOCH). FDG PET/CT performed after four cycles of DA-R-EPOCH showed very good response except for focus of disease in right abdomen.

**Figure 2:**
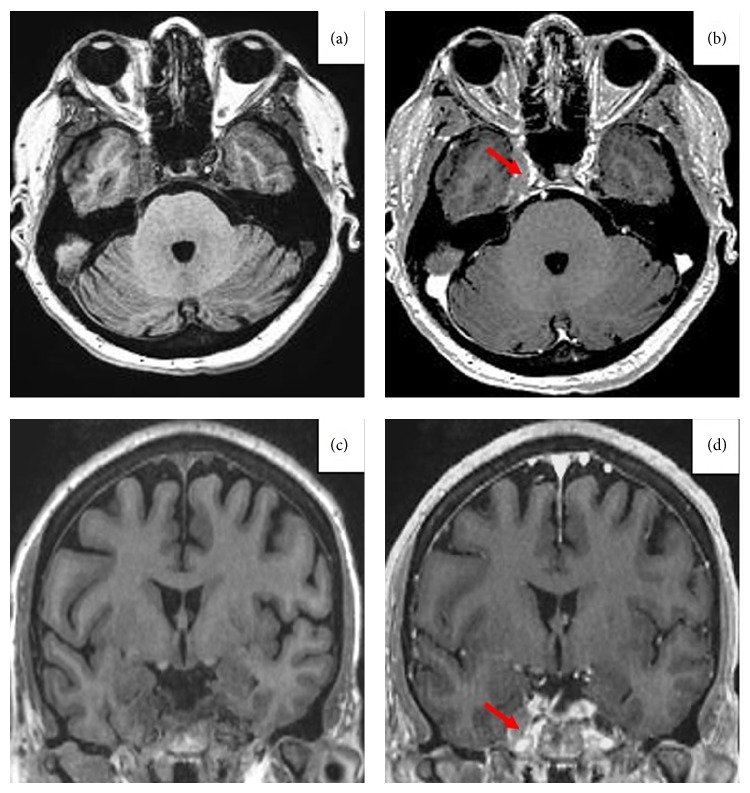
Transverse plane in T1W without (a) and with contrast (b) showing V nerve exiting the Gasserian ganglion (red arrow). Coronal plane in T1W without contrast (c) and with contrast (d) showing increased volume of the Gasserian ganglion in Meckel's cavum.

Three weeks after completing total planned 8 cycles of chemotherapy (one cycle of R-CHOP, seven cycles of DA-R-EPOCH, and seven cycles of intrathecal methotrexate for CNS prophylaxis), patient presented to the emergency department with worsening right eye pain, blurry vision, and diplopia. Physical examination of right eye revealed proptosis, sluggish pupillary light reflex, painful and restricted abduction, adduction, elevation, depression, and visual acuity of 20/70. Left eye exam was completely normal with visual acuity of 20/30. Decreased sensation to touch was noted in the distribution of the right first and second branches of trigeminal nerve. There was no temporal tenderness. Cranial nerves VII, IX, XI, and XII were intact on examination. There was no other focal neurological deficit. Slit lamp and fundus exam of both eyes were unremarkable. Magnetic imaging resonance (MRI) of brain with and without contrast showed swelling and enhancement of the right Gasserian (trigeminal) ganglion in Meckel's cave along with asymmetric prominence of the lateral margin of the right cavernous sinus. Magnetic resonance angiogram and cerebrospinal fluid analysis were normal. Painful ophthalmoplegia in the context of MRI findings affecting the right cavernous sinus and the Gasserian ganglion (Figure 2) and the absence of any other intracranial abnormality led to suspicion of Tolosa-Hunt syndrome (THS). Patient was started on high dose dexamethasone at 40 mg for 5 days with no improvement of symptoms. Repeat FDG PET/CT scan was done, which showed interval development of an FDG avid lesion along the lower aspect of left kidney that appeared to infiltrate perinephric region in the inferior aspect of the left lumbar fossa. Fine needle aspiration of perinephric mass confirmed the disease recurrence. Palliative radiation (1800 cGy) was administered to right posterior orbit and cavernous sinus. There was temporary amelioration of pain symptoms of right eye along with proptosis with no improvement in vision. Secondary to declining performance status, worsening ocular symptoms, and patient's wishes, hospice care was pursued. Patient expired soon after.

## 2. Discussion

Tolosa-Hunt syndrome (THS) is an idiopathic inflammatory condition that involves orbital apex and cavernous sinus manifesting in hemicranial pain, retroorbital or periorbital pain, loss of vision, oculomotor nerve paralysis, and sensory loss in distribution of ophthalmic and maxillary division of trigeminal nerve [1, 2]. This syndrome complex was originally reported in 1954 by Eduardo Tolosa and the eponym “Tolosa-Hunt syndrome” was applied in 1966 by Smith and Taxdal [3]. This idiopathic process is thought to be resulting from granulomatous inflammation within cavernous sinus (cavernous sinus syndrome) or orbital apex (orbital apex syndrome). The International Headache Society defines THS as episodes of steady unilateral orbital pain persisting for days to weeks, if untreated, resulting in paralysis of fourth and/or sixth cranial nerves, oculosympathetic paralysis, and spontaneous remissions and relapses with no demonstrable systemic disease outside the cavernous sinus [4]. Clinically this syndrome is simulated by multiple other disorders like trauma, vascular malformations, intracranial tumors, intracranial metastases, endocrine disorders, and inflammatory and infectious conditions [5]. To date, based on our review of literature there are only six reports of THS cases associated with lymphoma so far [6–11]. To the best of our knowledge this is the first case of DHL with cavernous sinus involvement mimicking THS.

Double-hit B cell lymphoma (DHL) is defined as diffuse large B cell lymphoma with translocations and/or extra signals involving MYC along with BCL2 and/or BCL6 as identified by FISH. In a large series with 129 patients diagnosed with DHL, it was noted that median age was 62 years (range, 18–85), 84% of patients had advanced-stage disease, and 87% had an International Prognostic Index (IPI) score ≥2 [12]. Complete response (CR), event-free survival (EFS), and overall survival (OS) were compared between R-CHOP, R-EPOCH, and R-HyperCVAD/MA (rituximab, hyperfractionated cyclophosphamide, vincristine, doxorubicin, and dexamethasone, alternating with cytarabine plus methotrexate) regimens. Multivariate analysis found lower CR in R-CHOP group compared to R-EPOCH group (odds ratio: 0.33, 95% CI 0.12–0.90, *P* = 0.031) or compared to R-Hyper CVAD/MA group (odds ratio: 0.30, 95% CI 0.22–0.79, *P* = 0.015). EFS and OS rates were also noted to be inferior in R-CHOP group compared to R-EPOCH group (*P* = 0.008 and *P* = 0.096). This study confirmed that the outcome of patients with DHL is poor with conventional chemotherapy. Salvage therapies are not clearly defined and do not seem to be effective with or without stem cell transplantation (SCT) suggesting that initial response to induction therapy is essential for long term survival. In another study of 39 cases from the Nebraska Lymphoma study group the median overall survival was only 9 months and the 5-year overall survival was 30%. Patients with low IPI scores of 0–2 had a better survival than those with high scores (3–5) [13]. In patients with DHL, higher coexpression of MYC (>30%) and BCL2 (50%) by IHC is found to be an independent predictor of poor survival [14]. In a recent multicenter survey of 311 patients treated at 23 academic North American centers, the PFS was 10.9 months and the OS was 21.9 months. Intensive induction improved PFS but not OS [15]. Autologous stem cell transplantation did not improve outcomes in patients who achieve complete remission [15]. Role of targeted agents in DHL is currently being explored and preclinical and clinical studies are underway testing PI3K inhibitors, Aurora kinase inhibitors, and BCL-2 inhibitors [16–18]. Phase II trial (NCT02272686) of Bruton's tyrosine kinase inhibitor (ibrutinib) after stem cell transplantation is also being considered [19]. Another phase I/II trial (NCT02213913) is also evaluating the role of lenalidomide when added to DA-R-EPOCH in patients with DHL [20]. The idea of evaluating therapeutic synergism using new targeted agents and traditional cytotoxic agents seems very exciting and promising. Only randomized trials could shed more light on this.

In summary Tolosa-Hunt syndrome is a rare clinical presentation and mandates thorough diagnostic workup to identify other causes that simulate it. In our patient, initial impression of Tolosa-Hunt syndrome, which was made based on clinical presentation and MRI findings, was soon realized to be secondary to lymphomatous infiltration from DHL which preceded systemic relapse as evidenced by FDG PET/CT scan. Lastly, in regard to DHL management, we recommended aggressive induction chemotherapy and participation in clinical trials when available and eligible.
